# The human photosensitive epilepsy model for clinical proof‐of‐principle trials of novel antiseizure medications. 1. Use of the EEG in drug development and characteristics of the model

**DOI:** 10.1111/epi.18468

**Published:** 2025-05-24

**Authors:** Dorothée Kasteleijn‐Nolst Trenité, Wolfgang Löscher

**Affiliations:** ^1^ Department of Neurosurgery and Epilepsy University Medical Center Utrecht Utrecht The Netherlands; ^2^ Nesmos Department, Faculty of Medicine and Psychology Sapienza University Rome Italy; ^3^ Translational Neuropharmacology Lab, NIFE, Department of Experimental Otology of the ENT Clinics Hannover Medical School Hannover Germany

**Keywords:** epileptiform discharges, genetics, intermittent photic stimulation, photoparoxysmal EEG response, proof of principle, reflex epilepsy

## Abstract

Clinical development of novel antiseizure medications (ASMs) would benefit from an early proof‐of‐principle (POP) model. The photosensitivity model, which uses the photoparoxysmal electroencephalography (EEG) response (PPR) as a surrogate for seizures, is currently the only human model that allows POP trials of investigational compounds after a single drug administration. Typically, trials in this model are performed as single‐blinded, placebo‐controlled Phase IIa POP studies, evaluating a range of doses in small groups of patients with epilepsy. Although most patients in such trials exhibit generalized epilepsies, photosensitivity also occurs in focal‐onset epilepsies. In the first part of this review, we describe the use of epileptiform discharges in drug testing, historical development of the photosensitivity model, the genetics and pathophysiology underlying the photosensitive response in patients with epilepsy, clinical characteristics of the patients, and details on drug testing. In the second part of this review, the outcome of numerous drug trials will be described in detail, including a critical discussion of the limitations of the model. In the past 50 years, the original and later standardized photosensitivity model has shown to be an unbiased, accurate, inexpensive method determining the potential efficacy of a novel ASM before entering large add‐on trials with chronic drug administration, irrespective of the type of epilepsy.

**Key points**

- Photoparoxysmal electroencephalography (EEG) responses (PPRs) are subtle ictal phenomena found in all types of epilepsy and at all ages.
- The mechanisms of photosensitivity are largely unknown but several candidate genes have been identified.
- Over the last 50 years, the human photosensitivity model has been standardized and validated for drug testing
- Antiseizure medications (ASMs) with diverse mechanisms of action are effective in suppressing PPRs.
- Drug testing in the photosensitive model is a useful tool to predict drug efficacy in both generalized and focal types of epilepsy.

## 1 INTRODUCTION

Photosensitive epilepsy is the most common subset of reflex epilepsy in humans.^1^, ^2^, ^3^, ^4^ Photosensitivity means literally abnormal sensitivity to light stimuli, where light or changes in light trigger visual disturbances, migraine attacks, and/or epileptic seizures.

In epilepsy, the term *photosensitivity* is used both for (1) epileptiform discharges evoked by intermittent photic stimulation (IPS) during an electroencephalography (EEG) recording and (2) epileptic seizures evoked by flickering (sun) light, TV, video games, and so on. Most patients with a clear history of seizures evoked by visual stimuli in daily life (visually induced seizures) have epileptiform EEG discharges during IPS, so‐called photoparoxysmal EEG response (PPR), if the entire stimulation procedure is conducted according to the consensus guidelines (see below).^5^, ^6^

Typical for photosensitive patients with epilepsy is sensitivity to flashing bright light, most commonly present with a flashing frequency between 10 and 30 flashes/s (Hz). Some patients—particularly untreated patients—are sensitive to light flashes between 2 and 60 Hz and are more vulnerable to environmental light changes than patients with a smaller range, for example, 20–25 Hz.^5^

In patients with epilepsy who are sensitive to flashing lights, epileptiform EEG discharges can be evoked with IPS repeatedly and reproducibly during wakefulness without evoking generalized tonic–clonic seizures if done with caution.^7^ PPR can be an activation of the preexisting epileptogenic area or a “stand‐alone” of generalized PPR in patients with epilepsy.^8^ Its occurrence is maximal between 10 and 20 years of age with clear female preponderance, although it is found at all ages and even in patients older than 50 years of age when there are no sex differences.^9^ Herein we use the term “photosensitive” to refer to patients with the photoparoxysmal EEG response correlate (PPR).

There are several misconceptions about photosensitivity and its use in drug evaluation. For example, there is a belief that the EEG PPR pattern is a generalized type of waveform seen *exclusively* in idiopathic generalized epilepsies (IGEs). That is not the case. PPR can occur in *all* types of epilepsy, including in focal‐onset and developmental and e*pileptic* encephalopathies, although it is more common in IGE.^2^, ^10^, ^11^, ^12^, ^13^, ^14^ A related myth is that the presence of a suppressive PPR effect by a new drug excludes (or is not necessarily indicative of) efficacy in focal‐onset seizures.^11^, ^15^, ^16^ Yet another common false suggestion is that the single‐dose IPS drug testing in photosensitive patients with epilepsy cannot accurately predict the effect and dosing of chronic administration of the drug in later clinical trials in patients with epilepsy.

In the first part of this review, we describe the historical development of the model, the genetics and pathophysiology underlying the photosensitive response, clinical characteristics of patients with photosensitive epilepsy, and details on drug testing in the model.

## 2 HISTORICAL DEVELOPMENT OF THE PHOTOSENSITIVITY MODEL

### 2.1 Use of the EEG in general

Epileptiform discharges on EEG are strongly related to epilepsy and are used for the diagnosis and evaluation of generalized and focal epilepsies. Routine EEG studies are often inter‐ictal. Since the early 1950s, clinicians and researchers have used the EEG to determine the effect of antiseizure medications (ASMs) in patients.^17^, ^18^ The most common finding of the ASM effect is the continuous and intermittent slowing of background waves. The effect of ASM on interictal epileptiform discharges (IEDs) is recognized, but the correlation between IEDs during baseline and after ASM intake has generally not been established due to variations in occurrence over time. Nevertheless, the effect on IEDs might give more useful information than background activities and spectral analyses of changes in background activities, so‐called quantitative EEG (qEEG).^19^

Some pharmacological EEG studies supported a relationship between suppression of IEDs and antiseizure efficacy of ASMs: in the 1980s, single‐dose drug studies in patients with drug‐resistant epilepsy were undertaken: benzodiazepines (BDZs) and phenytoin (PHT), given intravenously (i.v.) resulted in suppression of IEDs as well as acute seizure control.^20^ Lamotrigine (LTG; 240 mg) and diazepam (DZP; 20 mg) given orally, with spike‐counting for 10 min in 30 min epochs in a double‐blind randomized cross‐over study, resulted in significantly fewer IEDs 1 h after intake. No correlation could, however, be made between the plasma concentrations of LTG or DZP and IED counts.^21^ A double‐blind controlled study in 2001 on the effect of LTG in children with predominantly Lennox‐Gastaut syndrome showed both a reduction in the number and duration of IEDs longer than 30 s as well as a concomitant decrease in seizures.^22^ Another study with a retrospective analysis of the effect of LTG on the EEG of therapy‐resistant children and adults with a variety of seizure types showed fewer IEDs in parallel with clinical improvement.^23^

Overall, however, there exists little evidence of a direct correlation between antiseizure efficacy and IED suppression for most ASMs. Furthermore, only a few patients, such as those with Rolandic and classical absence seizures, have a high enough and stable frequency of IEDs to be eligible for those types of EEG studies. Even children with absence epilepsy show large variability in the localization and frequency of epileptiform discharges that might hinder studying drug effects on electrographic seizures on EEG.^24^

### 2.2 Use of the PPR

Several case reports of ASMs showed efficacy in seizure and PPR reduction in photosensitive patients since the 1950s: amobarbital, primidone, phenacemide, and para/tri‐methadione had a suppressive effect. In the 1960s, PHT, succinimides, phenobarbital, and DZP proved to be effective agents in this patient category.^25^

It was only in the mid‐1970s that the child neurologist Peter Jeavons from Birmingham, UK, who was interested in photosensitivity, studied together with his coworkers (Maheshwari and Clark, and later Covanis and Harding) the new French ASM sodium valproate (VPA) systematically and in a larger group of children with epilepsy (*n* = 63). Clinical and EEG improvement was impressive and seen in 87%, whereas only one patient showed clinical and EEG deterioration. Of the 17 photosensitive patients, 15 had a seizure reduction of at least 80%.^26^ Subsequently, other studies in photosensitive patients were performed in Birmingham, including a controlled study of the effect of VPA on patients with photosensitive epilepsy and its prognosis, in which the photosensitivity range—a measure of susceptibility to visually evoked seizures—was measured in 50 patients before and during prolonged treatment with VPA: photosensitivity on the EEG was reduced or abolished in 39 of the 50 patients.^27^ Following drug withdrawal, the photosensitivity range returned to its pre‐drug level. Of interest, they found a significant relationship between dose level in mg/kg and efficacy. VPA dose was higher in patients with seizure improvement or freedom (mean: 23.6 mg/kg; standard deviation [SD]: 6.74) than in those not significantly improved (mean: 17.4 mg/kg; SD: 4.32). Later studies from the same group confirmed the strong effect of VPA in 336 patients with a variety of epilepsy types (mostly IGE), including those with photosensitivity: 80% of the non‐photosensitive patients and 72% of the PPR‐positive patients became seizure‐free when VPA was given as monotherapy or added to carbamazepine (CBZ).^28^

These UK studies with standard IPS procedures engendered a strong belief that only VPA suppressed photosensitivity. Combined with other studies in photosensitive patients that emphasized the relationship between IGE and PPR, photosensitivity came mistakenly to be considered as *restricted* to IGEs. Similarly, the misconception arose that if a drug was shown effective in a proof‐of‐principle (POP) trial in patients with PPR, this limited its use to patients with photosensitive epilepsy only.^16^

Following VPA, levetiracetam (LEV) became the second choice after the impressive results of the POP trial in patients with photosensitive epilepsy in the 1990s.^29^ It has to be noted that various other ASMs became available in the years between the approval of VPA and LEV, but only a few (progabide, vigabatrin, LTG) were tested in the photosensitivity model and all were effective in suppressing the PPR (see second part of the review).

### 2.3 Development of the photosensitivity model with single‐dose and hourly range determinations

In the 1980s and 1990s, many novel potential ASMs were developed by the pharmaceutical industry and several of them were subsequently further characterized by the Anticonvulsant Screening Program (ASP) of the U.S. National Institutes of Neurological Disorders and Stroke (NINDS).^30^, ^31^ Human proof‐of‐efficacy (Phase II) trials were performed as add‐on trials in ASM‐resistant patients, were costly, and took at least 3 months to reach preliminary conclusions on drug effect, conclusions that were often misleading because of inherent weaknesses or bias in study design, analysis, and interpretation.^32^ Thus, there arose a need for early evidence of efficacy from short, small‐size studies before embarking on major pivotal trials.

Since the accidental discovery of naturally occurring reflex epilepsy with photosensitive seizures in a subspecies (*Papio papio*) of baboons from the Casamance region of Senegal in 1966 by Robert Naquet with his collaborators Eva and Keith Killam,^33^, ^34^ photosensitive baboons were considered a good genetic animal model for evaluating the efficacy and toxicity of novel ASMs.^35^, ^36^, ^37^, ^38^, ^39^, ^40^, ^41^ Accordingly, a photosensitivity human model was explored and developed in patients with photosensitive epilepsy.^42^ As a comparison between two EEG methods, single oral dose testing of LTG (240 mg) was done both by the half‐hourly spike‐count method and by hourly photosensitivity testing^43^: the size of the suppressive effect and time to effect overlapped, but a difference was found at baseline day, with the photosensitivity trial showing by far the most stable epileptiform discharges (PPR response ranges).

The photosensitivity human model with single‐dose administration of a new compound was then developed, utilizing the effect of a drug on IPS‐evoked generalized epileptiform EEG discharges. It was standardized with repeated IPS testing and quantification of responses unaffected by drowsiness—unlike other EEG outcome measures.^42^ The model has been further elaborated over the next decades into the current standardized adaptive Phase IIa POP trial design. Oral, intravenous or intranasal administration of the investigational compound can be evaluated, with PPR determinations, per hour, per 30 min, or per minute, in single‐ or double‐blind, placebo‐controlled study designs. Since then, the photosensitivity model has been applied successfully to various drugs (see second part of this review).

## 3 GENETICS AND PATHOPHYSIOLOGY OF THE PHOTOSENSITIVE RESPONSE IN HUMANS WITH EPILEPSY

The mechanisms of photosensitivity are largely unknown.^1^, ^44^, ^45^ As discussed below, several brain networks are involved in patients with generalized epilepsies; in patients with focal epilepsy, these networks might be different.

Screens of family members of people with photosensitive epilepsy indicate that the PPR is highly heritable, being inherited in an autosomal dominant fashion, with reduced and age‐dependent penetrance, indicating that multiple genes may contribute to photosensitivity.^1^, ^2^, ^46^ There have been several genome‐wide linkage studies looking for loci harboring candidate genes for the PPR trait in patients with genetic generalized epilepsies (GGEs).^4^ Among the genes that have been involved in PPR are ion channel genes, such as *TRPC4* (involved in calcium signaling) and *NEDD4‐2* (involved in the regulation of voltage‐gated sodium channels), as well as *BRD2*, which plays a role in chromatin remodeling and may influence neuronal activity and seizure susceptibility.^47^ Additional candidate genes are *GABR*_*A*_ *1* (encoding the alpha‐1 subunit of the γ‐aminobutyric acid (GABA)_A_ receptor), *CHD2* (involved in the regulation of transcription), and *SYNGAP1* (associated with glutamatergic neurotransmission).^1^, ^48^ Overall, the genetics of PPR exhibit significant heterogeneity, as it can manifest in various forms of epilepsy, including both genetic and non‐genetic syndromes. Of interest, in a chicken model of photosensitive epilepsy, a genetic animal model where the neurological disorder is inherited as an autosomal recessive mutation, a mutation in *SV2A*, the gene‐encoding synaptic vesicle glycoprotein 2A (SV2A), was found that causes an aberrant splicing event, significantly reducing the level of SV2A messenger RNA (mRNA) in homozygous carriers.^49^ The photosensitive epileptic chicken responded to the SV2A modulator LEV, suggesting that the low‐level expression of SV2A in these animals is sufficient to allow survival, but does not protect against seizures. Thus, SV2A is a very attractive candidate gene in the context of photosensitive epilepsy. A positron emission tomography (PET) ligand for SV2A has been examined as a marker of synaptic density in both baboons and humans,^50^ but, to our knowledge, it is not known whether SV2A binding of this ligand is altered in photosensitive baboons or patients (see also^51^).

The functional brain changes underlying PPR have been studied using EEG and magnetoencephalography (MEG), functional magnetic resonance imaging (fMRI), repetitive transcranial magnetic stimulation (rTMS), regional cerebral blood flow measures (RBF), and positron emission tomography (PET).^52^ Structural and metabolic changes have been identified using MRI techniques including MR spectroscopy (MRS). When the spatiotemporal distribution of PPR by EEG with implanted depth electrodes during presurgical assessment was studied, PPRs were characterized by activation of multifocal discharges in the parieto‐occipital, posterior cingulate, and medial prefrontal cortices, which reflects the cortical synchronization found in MEG and functional MRI (fMRI) studies.^52^ Differences in cortex volumes have been assessed by MRI voxel‐based morphometry (VBM). When compared with juvenile myoclonic epilepsy (JME) without photosensitivity, a reduced left hippocampus and left inferior frontal gyrus volume were observed among patients with photosensitive JME.^53^ Furthermore, the latter study showed reduced bilateral gray matter volume in the visual cortices using VBM in the JME‐photosensitive group compared to healthy controls. Comparative MRI studies revealed an increased bilateral thickness in the occipital, frontal, and parietal cortices in PPR‐positive subjects in comparison to healthy controls.^54^ Of interest, in photosensitive baboons with epilepsy, significant increases in gray matter volumes in the frontopolar, orbitofrontal, and temporal anterolateral cortices were found, whereas decreased volumes were observed in right primary visual cortices and the reticular, anterior, and medial dorsal nuclei of the thalamus.^55^ Although bi‐occipital resection eliminates the epileptic discharges in the naturally photosensitive baboon *Papio papio*,^56^ evoked potential studies in patients suggest that photosensitivity may result not only from occipital cortex hyperexcitability but also from diffuse or multifocal hyperexcitability involving cortico‐cortical pathways.^2^

Precipitation of seizures in photosensitive patients inevitably depends on the activation of a critical neuronal mass in the occipital cortex.^3^ Several observations indicate that the occipital cortex or visual system in patients with photosensitive epilepsy is hyperexcitable.^1^ Vaudano et al.^57^ found that PPR‐positive patients with GGEs had increased resting connectivity of the medial dorsal thalamus‐orbitofrontal cortex circuit, suggesting that photosensitivity is associated with less inhibition of networks that generate the alpha waves, the occipital EEG rhythm, that is connected with visual functions. Visual evoked potentials (VEPs) have a higher amplitude in photosensitive patients with generalized or occipital lobe epilepsies when compared to healthy controls or patients without photosensitive epilepsy. In addition, VEPs in patients with photosensitive epilepsy respond differently to rTMS than VEPs in controls in that VEPs in patients with photosensitive epilepsy recovered faster after rTMS, which may correspond to the tendency to have photosensitive seizures.^58^ However, as described above, abnormal excitability in patients with photosensitive epilepsy extends beyond the occipital cortex and visual system.^1^, ^3^

Often the assumption is that photosensitive seizures are generalized in onset. However, we concluded previously from a literature review that 17% of focal epilepsies show PPRs and that PPRs can be focal.^11^ Other groups observed PPR in 20% of patients with focal seizures.^12^, ^59^ PPR has been found in patients with occipital epilepsy and temporal lobe epilepsy.^60^ Substantial evidence exists for photosensitive seizures originating focally near the visual cortex.^61^ In this respect, it is interesting to note that evidence from animal, clinical, neurophysiological, and neuroimaging studies support the concept that “generalized” reflex seizures, usually occurring in the setting of IGE, should be considered as focal seizures with quick secondary generalization.^2^ Overall, as discussed in the second part of this review, PPRs can definitely be used to evaluate the efficacy of new ASMs for patients with focal‐onset epilepsies.

## 4 CLINICAL CHARACTERISTICS OF PATIENTS WITH PHOTOSENSITIVE EPILEPSY

### 4.1 Signs and symptoms evoked by IPS and clinical history

**FIGURE 1:**
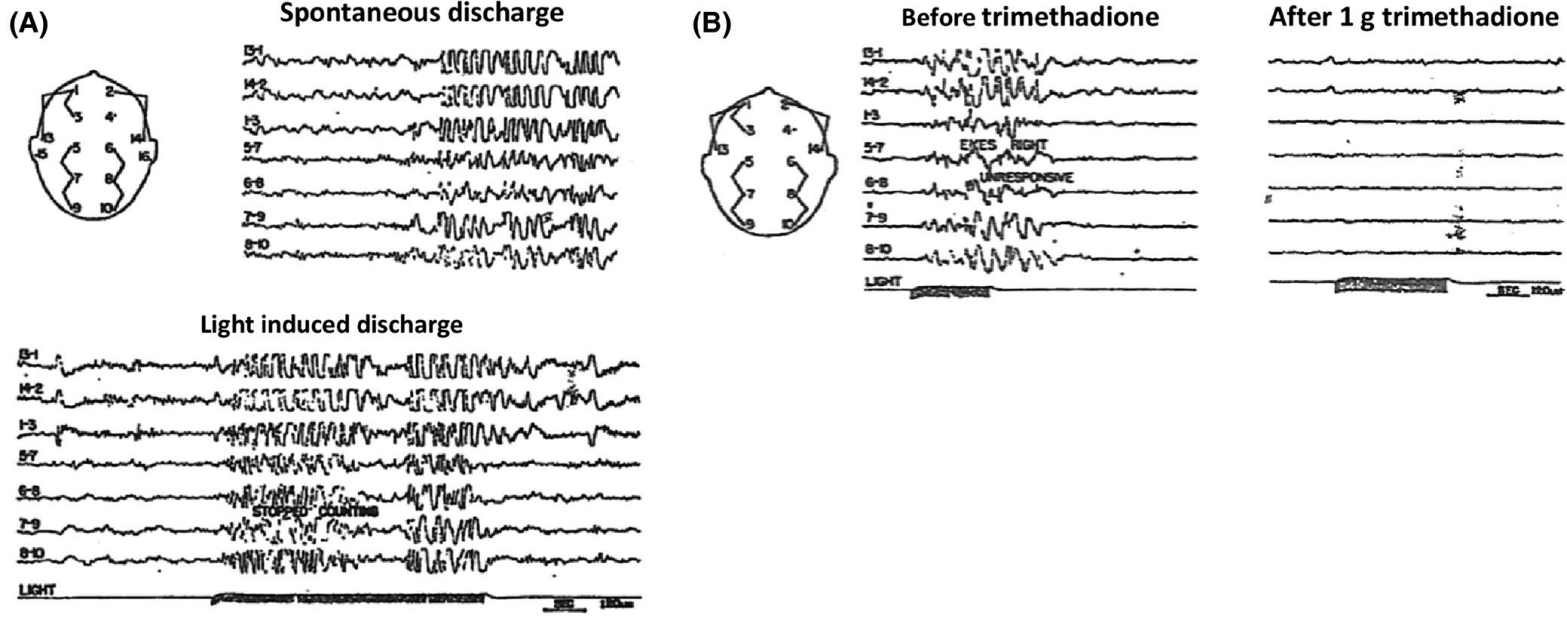
The photoparoxysmal electroencephalography (EEG) response (PPR) as a biomarker of epilepsy and effects of treatment. (A) Similarity between spontaneous and intermittent photic stimulation (IPS)–evoked epileptiform discharges indicates the PPR as a biomarker of epilepsy. (B) Suppressive effect 2 min after 1 g trimethadione intravenously on the PPR evoked by intermittent photic stimulation. From Bickford et al.^62^

As early as 1953, a relation between PPRs and clinical signs and symptoms was noticed in a detailed study of 27 children with epilepsy^62^: eyes turning to the left or right; head‐turning; myoclonic jerking of the head, limbs, or eyelids; opening of the eyes; speech arrest; feelings of dizziness or none. Bickford et al.^62^ also noticed a variety of electroencephalographic variants and a suppressant effect of trimethadione, amobarbital, and phenacemide (Figure 1). These drugs (i.v. or oral) reduced or suppressed the PPRs and in parallel their clinical symptoms. Based on these observations, the authors proposed the following classification that would be (and to date is) of value for clinicians: (1) a clinically sensitive group in which light of the intensity encountered in daily life is capable of inducing clinical attacks (5 children); (2) a less‐sensitive group in which clinical seizures can be induced only under conditions of high intensity of illumination and rapid flicker, which can be produced in the EEG laboratory (15 children); and (3) a group in which the only evidence of sensitivity is the occurrence of EEG discharges in relation to stimulation by light and unaccompanied by any detectable clinical evidence of seizure (7 children). Patients can change from one group to another, either by lowering their threshold or increasing their threshold through an ASM.

In a similar detailed type of study in 36 patients (19 F; mean age 18 years, range 4–41; 13 ASM free) with generalized epilepsy in 81% (idiopathic 53%) and focal epilepsy in 17%,^63^ it appeared that clinical features could be observed in 72% of those with a PPR of .5–3 s duration. Only about half of the patients, however, noticed the evoked myoclonic jerks, which explains that patients often have difficulty in recognizing visually evoked seizures in daily life. This number increased if patients had symptoms of ocular discomfort (69% vs 25% in the non‐photosensitive age‐matched control group). Pain in the eyes was very specific for the photosensitive patients. In 60%, the history of seizures was concordant with the observed clinical features during IPS.

When acute behavioral changes in simple motor response were measured during subclinical PPR, *all* children showed a delay and thus, as the authors stated, PPR represents provoked seizures.^64^ An Australian study investigated the importance of the finding of PPR in routine EEG diagnostics: 8% (21/263) of children had mainly brief/self‐limited PPRs, which correlated with epilepsy (76%).^65^

In a recent study in patients with GGE, a comparison of PPR and spontaneous seizure morphology found them indistinguishable, suggesting that PPR is a valid proxy for seizure dynamics.^66^

### 4.2 PPR in focal epilepsies?

It is increasingly recognized that focal EEG abnormalities and ictal semiology can occur in generalized epilepsies, and absence seizures can be associated with focal epilepsies.^67^, ^68^, ^69^ Gilliam and Chiappa^70^ investigated the relation between spontaneous epileptiform abnormalities and PPR in 115 IPS‐positive patients with an age range of 3–79 years (median 22.5): 41 had generalized spontaneous discharges, 17 only focal, and 10 both focal and generalized ones. A statistically highly significant association was found between the type of spontaneous discharges and seizure classification. Patients with focal spontaneous discharges have nearly always focal seizures and those with generalized spontaneous discharges, generalized seizures. PPRs are found in patients with occipital and temporal lobe epilepsy, even with ictal focal seizure symptomatology during the PPR.^60^, ^71^, ^72^, ^73^ Nicolson et al.^74^ described seven patients with both focal (epigastric, gustatory, déjà‐vu, and one with visual aura) and generalized epilepsy: all showed generalized spike‐waves and two showed spikes over the temporal region. Six of these seven patients showed generalized PPRs. One patient with a left temporal focus and frequent focal seizures evolving into generalized tonic–clonic seizures (GTCS) was operated (left‐sided amygdalo‐hippocampectomy) and mesial temporal sclerosis was confirmed; she became and remained seizure‐free, also after discontinuation of her ASMs.

Based on information from a variety of studies and close observations, one can conclude that PPRs are an ictal phenomenon found in all types of epilepsy and at all ages associated with a variety of clinical symptoms and signs related to the type of epilepsy.^9^ The strongest expression of PPR (greater range and predominantly associated with myoclonic jerks) is in females aged 10–30 years with generalized (idiopathic and encephalopathic) epilepsies.

PPR can thus be used as a surrogate marker of epilepsy, indicating increased cerebral excitability. As such, it is well suited for the clinical screening of potential ASMs.

### 4.3 Effect of ASM treatment

In the past few decades, clinical Phase II and Phase III pivotal add‐on studies for drug approval or Phase IV post‐marketing studies of new ASMs did not include EEG analyses.^75^ Only the POP photosensitivity studies and a few qEEG analyses used the EEG as an outcome measure of efficacy. Some cases are described that showed suppression of the PPR in combination with clinical improvement, for instance, with lacosamide and zonisamide in JME and Unverricht‐Lundborg disease, respectively.^76^, ^77^

The ASMs tested in the clinical photosensitivity model over the years and found to be effective in suppressing the PPR with a single dose, all showed efficacy in multiple‐dose Phase II/III clinical trials and clinical practice (see second part of this review). It is important to note that not all patients show a PPR suppressant effect in the model, as is the case with drug‐resistant seizures in clinical practice. Jeavons and Clark^26^ studied the effect of VPA in 63 patients with various types of epilepsy; similarly they found in a subgroup of 17 photosensitive patients different degrees of suppression, whereas one patient showed no effect at all. Acute dosing of VPA in patients with photosensitive epilepsy by Rowan et al.^78^ suppressed PPR (completely or substantially) in seven of nine patients. Of interest, the effect appeared 1–5 h after attainment of peak VPA plasma levels and lasted up to 5 days. A delayed onset and carry‐over effect of VPA were also reported in animal experiments and may, at least in part, be related to the formation of the active metabolite, trans‐2‐en‐VPA.^79^, ^80^ Binnie et al.^42^ reported that VPA reduced or abolished PPR in 5 of 8 patients with 600 mg and 6 of 10 patients with 900 mg/kg; that is, about 40% of the patients did not respond to VPA. This may suggest that the photosensitivity model predicts not only the antiseizure efficacy of ASMs but also drug resistance. However, as discussed in the second part of this review, this assumption is not true.

**FIGURE 2:**
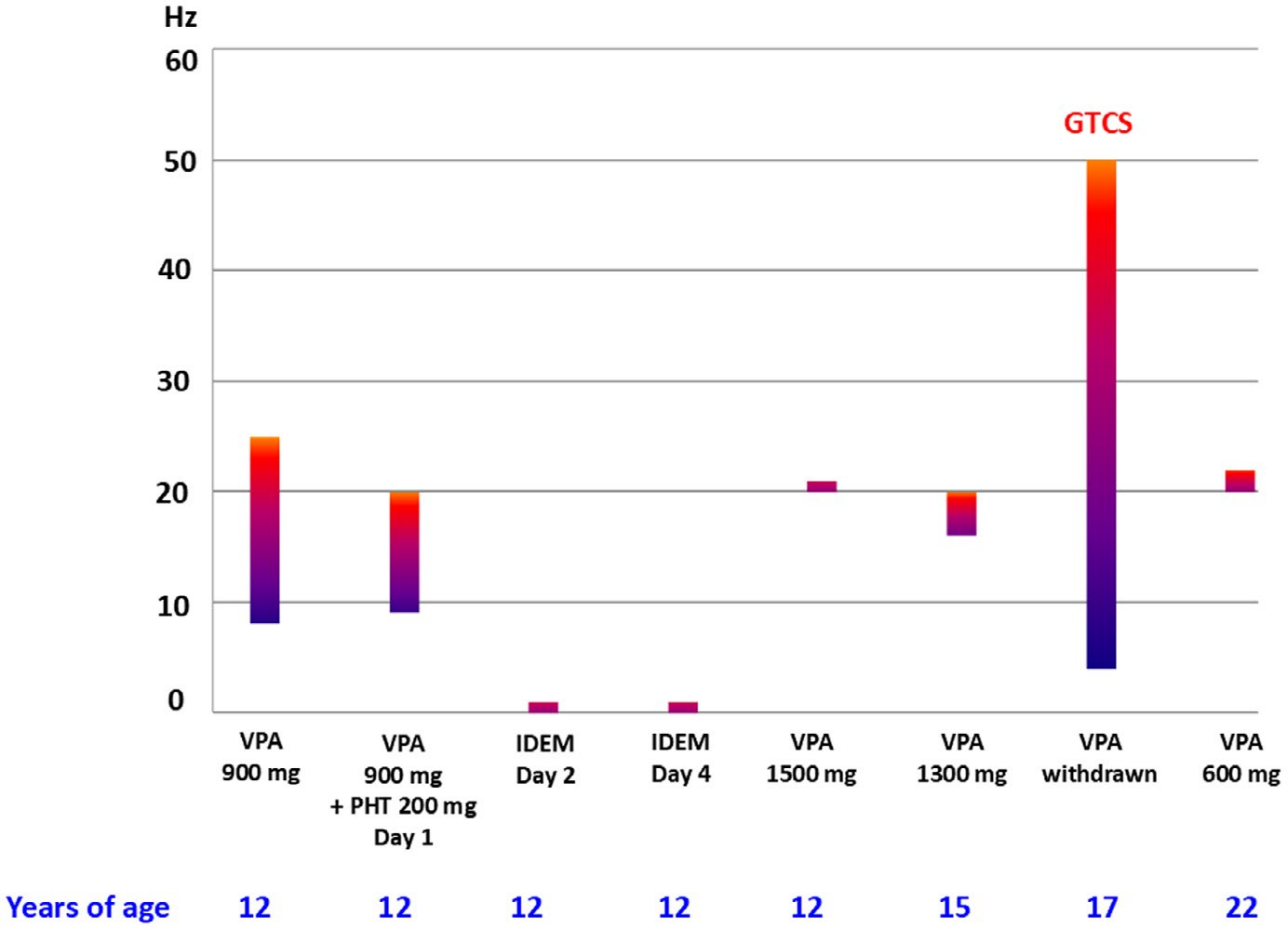
Effect of valproate (VPA) withdrawal on the photosensitivity range and seizure recurrence in a female patient with photosensitive genetic generalized epilepsy. The patient was treated with VPA over ~10 years and the photoparoxysmal electroencephalography (EEG) response (PPR) was repeatedly determined by intermittent photic stimulation (IPS) as shown in the figure. When the patient was 12, phenytoin (PHT) was added to VPA over several days because VPA (900 mg) alone did not completely abolish the PPR; the drug combination completely suppressed the PPR (and controlled the seizures). Later, it was considered better for the patient to withdraw PHT due to side effects, whereas the dose of VPA was increased to 1500 mg, which almost completely suppressed the PPR (and controlled the seizures). When the patient was 17, that is, after 5 years of suppression of PPR and seizures, VPA was withdrawn, leading to a large PPR and the occurrence of generalized tonic–clonic seizures (GTCS). Reinstallment of VPA treatment led again to an almost complete abolishment of PPR and the disappearance of clinical seizures over subsequent years. *Source*: Unpublished data from D. Kasteleijn‐Nolst Trenité.

The number of flash frequencies (so‐called range) that evoke a PPR is related to clinical parameters,^5^ and complete PPR suppression is not necessary for a good clinical outcome. An example is given in Figure 2. This example also illustrates the risk of ASM withdrawal in patients with photosensitive epilepsy and the potential role of IPS in monitoring ASM withdrawal.

## 5 THE METHODOLOGY OF DRUG TESTING IN THE PHOTOSENSITIVITY MODEL

Clinical ASM development would benefit from a human model permitting early demonstration of antiseizure activity with short‐term dosing, ideally after a single dose.^81^ The photosensitivity model has been used successfully as a preliminary assessment of efficacy (dose–response) and tolerability of single doses of potential new ASMs in epilepsy patients in early Phase II studies during the past ~50 years (see second part of the review). Drug testing consists of repeated standardized determination of photosensitivity ranges, as measured with the EEG response to IPS at a fixed number of flash frequencies, before and after intake of a single dose of the drug under investigation (see schematic presentation in Table 1). The trial lasts 3 days with a similar EEG–IPS protocol during those days: the first day is baseline (placebo control), the second day is drug day with administration of a single dose of the new compound after another baseline measurement before the drug administration, and the third day is used for determination of duration of the PPR suppressive drug effect. Every IPS series (every hour) is combined with blood sampling to make a pharmacokinetic(PK)/pharmacodynamic(PD) correlation as close to each other as possible.

**TABLE 1:** This table shows the design of the classical photosensitivity model.

	Screening	Refresh	Assess	Assess	Assess	Assess	Assess	Assess	Assess	Assess	Final visit day 3/4
Day	−21 until −1	1, 2, 3 and optional 4									
Time (hours from baseline)	Afternoon	≥−2	0	+1	+ 2	+3	+4	+5	+6	+8	
Signed informed consent	✗										
Inclusion/exclusion	✗		✗								
Pregnancy test	✗		✗								✗
Medical history	✗										✗
Physical examination	✗										✗
Administration of placebo or drug X			✗								
EEG‐IPS assessments	✗		✗	✗	✗	✗	✗	✗	✗	✗	
DRUG X samples			✗	✗	✗	✗	✗	✗	✗	✗	
ASM blood samples	✗		✗	✗	✗	✗	✗	✗	✗	✗	
Adverse event monitoring	✗		✗	✗	✗	✗	✗	✗	✗	✗	✗

*Note*: Patients who are taking ASM comedication will continue their antiseizure medication (ASM) exactly as prescribed in order not to disturb their brain equilibrium. Adaptation of timing of drug intake (e.g., intervals of 15 min for the first 3 h) are possible as long as the photoparoxysmal response (PPR) determinations are scheduled at exactly the same times during all investigational days and in synchrony with the blood samples taken. Psychological tests and questionnaires could be performed in‐between the intermittent photic stimulation (IPS) sessions as deemed valuable. Per patient, the same trial performer and location is important because results will be compared between the experimental days in the same patient, so the patient serves as his/her own control.

The safety of patients is monitored by personal and video observation and regular physical and neurological examinations, and a protocol that stops photic stimulation when positive PPR occurs so as to minimize the risk of a seizure. After the first two to four patients, a decision is made if the next dose can be higher or lower in order to have a good dose–response relationship profile of the new compound. Per dose, four patients are commonly considered sufficient (see Part 2 for further statistical explanation).

The design allows adaptation in number and timing of IPS testing (so‐called adaptive trial design), dependent on the expected onset of effect and maximal dose, but above all on questions that need to be answered based on (single dose) data gathered in animals and healthy volunteers.

### 5.1 Patient selection

Eligible are all types of epilepsy patients that show a reproducible IPS‐induced PPR on EEG. Patients can stay on concomitant medication (restricted though to two ASM per protocol) as prescribed. They are their own control. The type of epilepsy is therefore also less relevant.

### 5.2 Determination of the pharmacodynamic outcome parameters

**FIGURE 3:**
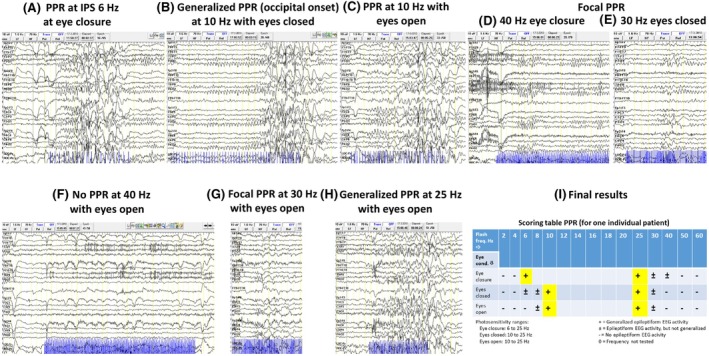
Examples of the determination of the lower and upper sensitivity thresholds per eye condition. All examples are from the same patient with photosensitive epilepsy (female, 23 years of age). As shown in I, the upper thresholds for eye closure and eyes closed are in this patient similar to the eyes open condition. In Section 5.2 the methodology of IPS stimulation is described. Photic stimulation at the moment of eye closure on demand is the most sensitive and also stable condition. There are, however, individual differences.

In a dimly lit room, EEG‐IPS (standard 21‐channel EEG equipment with high‐quality photo stimulator) is performed,^82^ while the patient is seated to allow proper observation of the eye conditions during photic stimulation as well as possible occurring clinical signs. Flashing is one per flash frequency for 5 s at max duration with at least 5 s pause and immediate stop of the stimulus when generalized epileptiform discharges are seen on EEG. Both lower and upper limits or thresholds of sensitivity, as expressed by a generalized PPR—the so‐called range or threshold limits—are determined by using flash frequencies in the following order: 2, 4, 5, 8, 10, 13, 15, 18, 20, 23, 25, 30, 40, 50, and 60 Hz. As soon as a generalized PPR occurs, the next flash frequency starts at 60 Hz while decreasing the flash frequency until again a generalized PPR is seen. These photosensitivity ranges (pharmacodynamic measures) are measured in three separate eye conditions: eye closure, eyes closed, and eyes open. An example of an individual patient is shown in Figure 3. Starting IPS at the same time as eyes closing on demand (“eye closure”) has proven to be more provocative than stimulation during eyes closed or open.^5^

### 5.3 Analyses

**FIGURE 4:**
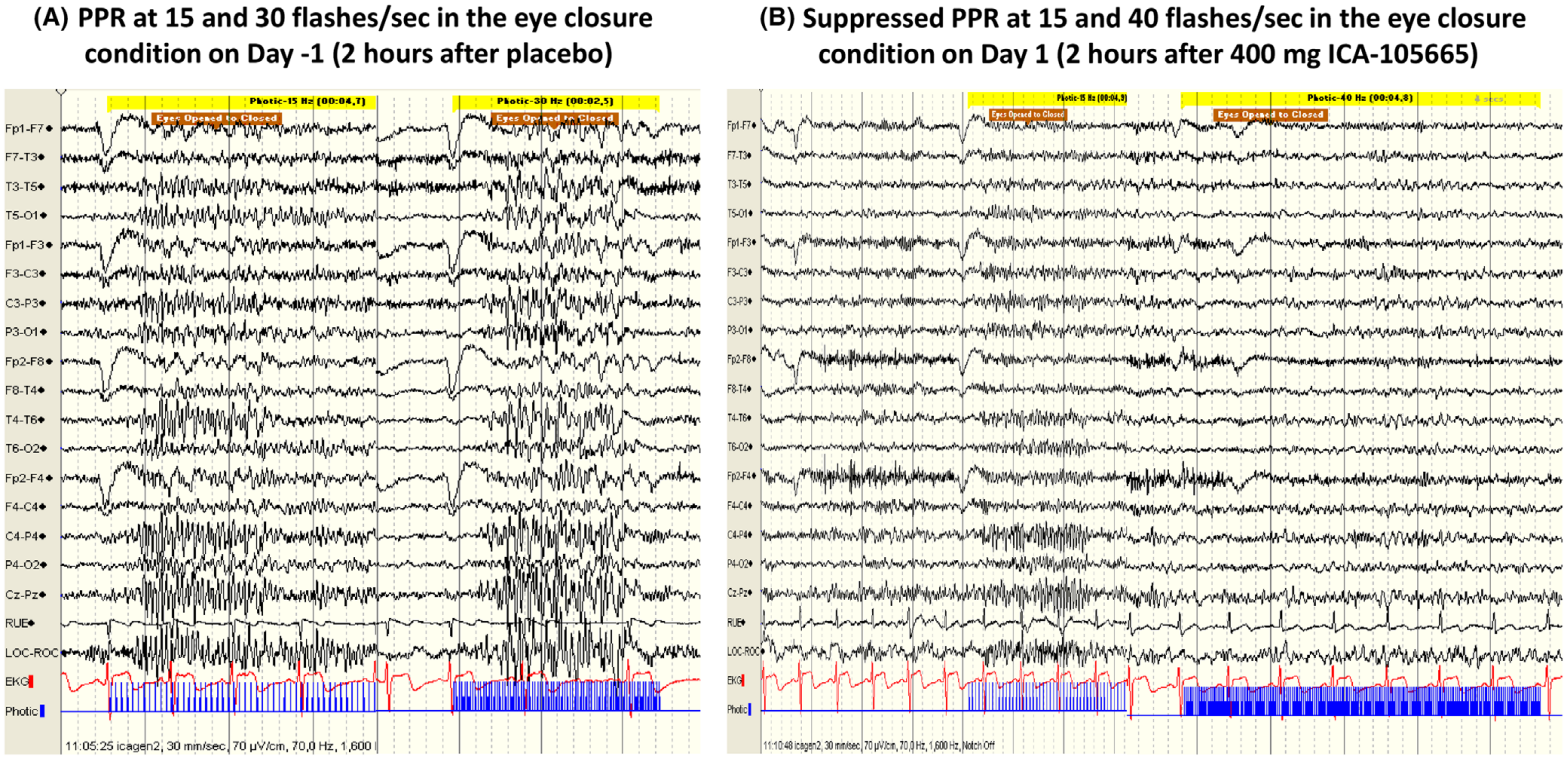
Example of an adult photosensitive epilepsy patient with PPR at 15 Hz and 30 Hz or 40 Hz (eye closure condition), first (A) 2 h after placebo (Day −1) and then (B) 2 h after intake of the experimental drug, which suppressed the PPR (Day 1). The drug in this example was the KCNQ potassium channel activator ICA‐105665.

A central blinded reader of all EEG studies and patients blinded to the study treatment make sure that the data are robust for further analyses. The determined lower and upper thresholds in Hz (ranges) for each patient test are transformed into another metric, called the standardized photosensitive range (SPR). This means that with a maximum of 15, based on the total number of flash frequencies tested, a range of 13–30 Hz corresponds to a SPR of 7. Suppression of the range is defined as at least suppression of three frequency levels (SPR of 3) of the maximum of 15, in at least one eye condition on the SPR scale, or complete abolition of the PPR. Furthermore, the onset time of reduction in SPRs and duration of the observed changes (period of reduction of SPRs) are determined. Every patient serves as his/her control. Paired *t* tests or other more sophisticated statistical analyses are done per patient as well as for the total sample per predetermined time period, to see the effect size over time after intake of the drug (see the published POP trials that used the model; described in the second part of the review). A robust effect is considered if 75% of the patients of a particular dose group had a suppression of the EEG response to IPS after intake of the compound on Day 1 vs baseline measurements while on placebo on Day −1. An example of PPR in the EEG of an individual patient before and after drug intake is shown in Figure 4.

**FIGURE 5:**
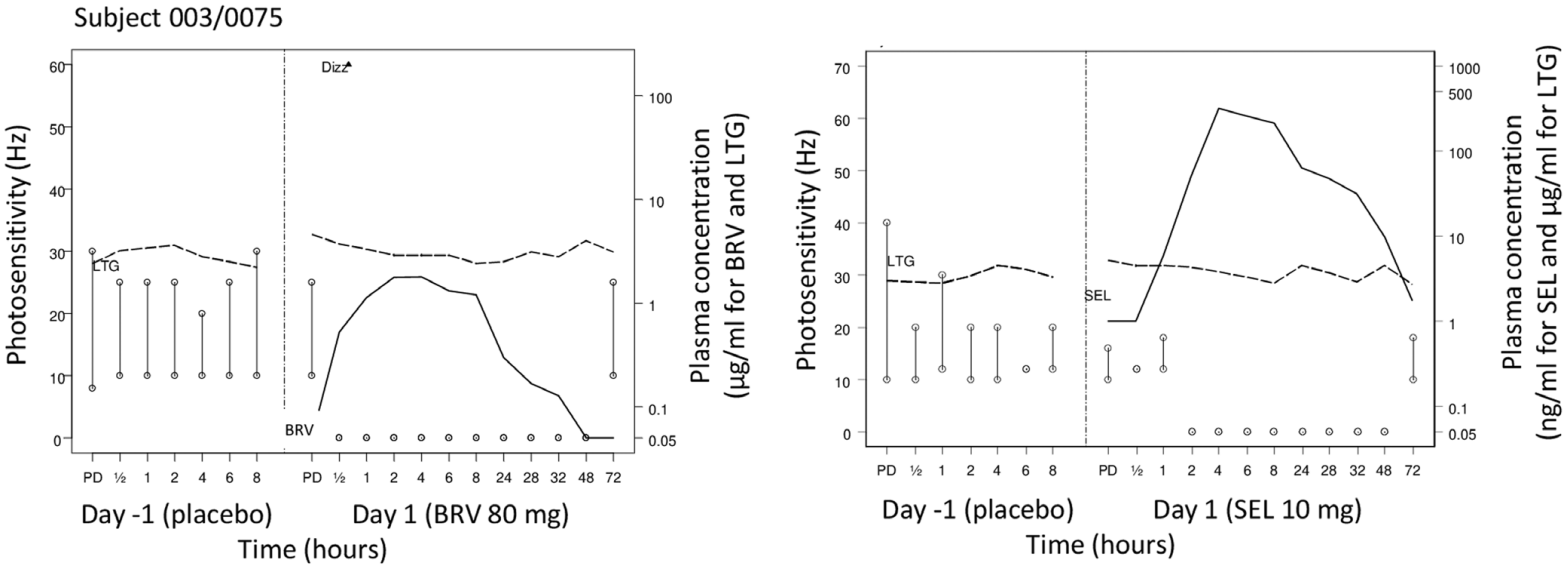
Example of an individual patient with photosensitive epilepsy who was treated with brivaracetam (BRV) and seletracetam (SEL) in different trials with an inter‐trial interval of ~1 year. In each trial, the patient received a placebo (Day −1; left part of each graph) and BRV or SEL (Day 1; right part of each graph). “PD” = “predrug.” The effect of oral intake of BRV and SEL on the photosensitivity range (upper and lower photosensitivity limits, in Hz depicted as a bar) is shown. The limits themselves are graphically expressed as small circles. The left y‐axis shows the photosensitivity in Hz, whereas the right y‐axis shows the plasma drug concentration of BRV (in μg/mL), SEL (in ng/mL), or the antiseizure medication (in μg/mL) that the patient received on a chronic basis. The patient (Subject 003/0075) shown here was pretreated with lamotrigine (LTG) in both trials. Drug levels are indicated by the stippled (LTG) or solid (BRV, SEL) lines. Both BRV (80 mg) and SEL (10 mg) completely abolished the photoparoxysmal response. Note the appearance of dizziness after BRV but not SEL. Modified from Kasteleijn‐Nolst Trenité et al.^83^

Finally, all PK and safety data are combined in a graph per patient and per dose. This allows maximal information on the new compound to make decisions on further trials. Figure 5 shows an example of the outcome parameters of a patients with photosensitive epilepsy who was treated with brivaracetam (BRV) and the investigational drug seletracetam (SEL) in two separate trials.

## 6 CONCLUSIONS

Over about 50 years of experience with drug testing in the photosensitivity model, a standardized adaptive Phase IIa trial protocol has been developed and validated with approved ASMs. This model has subsequently been used for POP trials with numerous investigational compounds in patients with photosensitive epilepsy. The outcome of these studies are described in detail in the second part of this review, including a critical discussion of the limitations of the model.^84^

## CONFLICT OF INTEREST STATEMENT

D. Kasteleijn‐Nolst Trenité has received in the past 5 years consultancy fees from UCB, Otsuka, SK, Idorsia, Lundbeck, Jazz, Praxis, Axonis, and NeuroPro. She also receives royalties from Wolters Kluwer (UpToDate). W. Löscher is cofounder and CSO of PrevEp, Inc. (Bethesda, MD, USA). He has received in the past 5 years consultancy fees from Lundbeck, Angelini, Clexio, Selene, Axonis, SynapCell, Sintetica, ND Capital, Atlas Venture, Cogent Biosolutions, Ovid, Idorsia, and Addex.

## ETHICS STATEMENT

We confirm that we have read the Journal's position on issues involved in ethical publication and affirm that this report is consistent with those guidelines.

## Data Availability

No primary data were collected for this study. The original data extraction is available from the corresponding authors upon reasonable request.
